# Energy balance in patients with advanced NSCLC, metastatic melanoma and metastatic breast cancer receiving chemotherapy – a longitudinal study

**DOI:** 10.1038/sj.bjc.6602357

**Published:** 2005-02-22

**Authors:** M N Harvie, A Howell, N Thatcher, A Baildam, I Campbell

**Affiliations:** 1 Cancer Research UK Department of Medical Oncology, Christie Hospital, Wilmslow Road, Manchester M20 4BX, UK; 2 University Department of Surgery, South Manchester University Hospitals NHS Trust, Manchester M20 2LR, UK; 3 University Department of Anaesthesia, South Manchester University Hospitals NHS Trust, Manchester M20 2LR, UK

**Keywords:** metastatic, body composition, energy balance, acute-phase protein response, chemotherapy, longitudinal

## Abstract

Chemotherapy exerts a variable effect on nutritional status. It is not known whether loss of body fat or fat-free mass (FFM) during chemotherapy relates to diminished dietary intake, failure to meet elevated energy requirements, or to the presence of an acute-phase response. We sought to determine prospective measurements of body mass and composition, resting energy expenditure, energy and protein intake, and C-reactive protein over a course of chemotherapy in 82 patients with advanced cancer. There was a large dropout from the study. Prospective measurements were obtained in 19 patients with non-small-cell lung cancer (NSCLC), 12 with metastatic melanoma and 10 with metastatic breast cancer. There were significant increases in energy intake among patients with metastatic breast cancer, 873 (266–1480) kJ (mean 95% CI; *P*<0.01), and metastatic melanoma, 2513 (523–4503) kJ (*P*<0.01). Breast cancer patients gained percentage body fat over the course of treatment, 2.1 (0.8–3.5%). Gain or loss of body fat correlated to mean energy intake throughout chemotherapy in patients with NSCLC (Rs=0.751; *P*<0.01) and metastatic breast cancer (Rs=0.617; *P*<0.05). The ability to meet or exceed energy requirements led to gains in body fat among patients with metastatic breast cancer and NSCLC, but did not prevent loss of FFM in these groups.

Treatment with chemotherapy is linked to loss of weight (i.e. loss of body fat and/or fat-free mass (FFM)) in some, but by no means all, cancer patients (Jardine *et al*, 1984; Jebb *et al*, 1994; Sarna *et al*, 1994). Whether loss of body fat and FFM are related to diminished dietary intake or a failure to meet the elevated energy requirements due to increases in resting energy expenditure associated with cancer and its treatment is not resolved, as there are few prospective data of energy balance among patients receiving chemotherapy. A further consideration is the role of the acute-phase response. This has been linked to weight loss through the associated elevations in REE (Staal-van den Brekel *et al*, 1995) and declines in dietary intake (Wigmore *et al*, 1997), but may also specifically lead to the depletion of FFM (Simons *et al*, 1999). The aim of this study was to examine whether changes in dietary intake, resting energy expenditure or the acute-phase response could explain changes in weight and body composition over the course of chemotherapy. We therefore measured body mass, body composition, energy expenditure, energy intake and C-reactive protein (CRP) over several cycles of chemotherapy among patients considered to be at high (non-small-cell lung cancer (NSCLC)) (Buccheri and Ferrigno, 2001) and metastatic melanoma (Smit *et al*, 1983), and low (metastatic breast) risk of wasting (Jardine *et al*, 1984).

## PATIENTS AND METHODS

### Patients

A total of 105 patients with advanced NSCLC (stage 111 or IV), 40 with metastatic melanoma and 30 with metastatic breast cancer and scheduled to have chemotherapy were invited to enter the study. None of the patients had received any cancer therapy, that is, surgery, chemotherapy, radiotherapy or endocrine therapy within the previous 3 months. Patients with a prognosis of less than 2 months or severe endocrine abnormalities (e.g. diabetes mellitus, or hyper/hypothyroidism) were excluded. None of the patients received active nutritional intervention. Tumour response to chemotherapy was evaluated according to the standard criteria for melanoma, NSCLC (World Health Organisation, 1979) and breast cancer patients (UICC, 1987).

### Measurements

Patients were seen before commencing chemotherapy, prior to the second chemotherapy cycle, and 1-month post completion of chemotherapy (4–6 cycles). On each occasion, the following measurements were determined:

*Body and FFM*: were determined from weight and skinfolds (biceps, triceps subscapular, suprailiac sites) using the equations of Durnin and Womersley (1974). Skinfolds were measured three times by a trained individual and the mean values calculated. The coefficients of variation (CVs) for the determination of fat and FFM from skinfolds by MH on different occasions were 1.1 and 0.8%.

*Mid-arm muscle circumference (MAMC)*: was calculated from tricep skinfold (TSF) and mid-arm circumference using standard equations (Gurney and Jelliffe, 1973). The CV for the determination of MAMC measured by MH on different occasions was 1.5%.

*Total body potassium*: Total body ^40^K (TBK), a measure of lean body mass, was measured using a NE8108 shadow shield whole-body monitor (Nuclear Enterprises Ltd, Edinburgh, UK) over 40 min. Relative changes only in ^40^K were determined. No attempt was made to perform absolute measurements, as this would have involved administration of radioactive isotopes. Sensitivity of the detectors and instrumental stability were determined after each measurement by performing a static count of potassium standard. The measured values were corrected for changes in sensitivity of the scanner and any change in body size of the patient between measurements using a correction factor previously described (Boddy *et al*, 1971). Coefficient of variation of measurement was 2%.

*Energy and protein intakes*: were determined from four-day weighed food diaries completed before each assessment in the week prior to the next chemotherapy cycle to avoid the acute effects of the treatment on dietary intake. Energy intake was determined using the Compeat 4 Nutritional Analysis System (Carlson Bengston Consultants, London) based on the 5th Edition of McCance and Widdowson The Composition of Foods (Holland, 1991).

*Resting energy expenditure*: was determined by indirect calorimetry (Deltatrac Metabolic Monitor MBM 100, Datex Instrumentation Corporation, Helsinki, Finland) under standardised conditions (Harvie *et al*, 2004). Oral temperature was recorded using the 3M Tempa-DOT Thermometer (3M Health Care, USA) calibrated against a National Physics Laboratory thermometer to confirm that patients were apyrexial, and had no overt signs of infection. Resting energy expenditure was also determined in healthy men and women recruited from hospital staff who were one-to-one sex and age matched to the three different cancer subgroups to control for this measurement.

*Serum CRP*: concentration was measured using a turbidometric method with specific antibodies (Dako Patts, Denmark), measuring the change in absorbance at 340 in an automated analyser (Cobas Mira, Roche). Concentrations were determined using a multi-point calibration curve (Hellsing, 1973) at a CRP concentration of 35 mg l^−1^. Coefficient of variation was 8.6%.

*Performance status*: of each patient was evaluated prior to chemotherapy by assigning a Karnofsky score based on the ability to carry out normal activities (Karnofsky and Burchenel, 1949).

*Health-related quality of life*: was assessed over the week prior to each visit using the self-report general Functional Assessment of Cancer Therapy Scale (FACT-G) (Cella *et al*, 1993). This 28-item questionnaire assessed physical, functional, emotional and social well-being. Higher scores are associated with better function, with a maximum total score of 116.

### Ethics

The study was approved by the South Manchester University Hospitals NHS Trust Ethics Committee. All participants provided written informed consent.

### Statistical analysis

These were performed using the Statistical Package for Social Sciences (SPSS 2001 version 10.1.4). Significance was accepted at 0.05 level of probability. Resting energy expenditure in patients with advanced NSCLC, metastatic melanoma and metastatic breast cancer were compared to healthy controls using the independent *t*-test.

Changes in outcome variable between baseline and the end of chemotherapy were determined in each of the groups from paired *t*-test (weight, body fat, % fat MAMC TBK, FACT-G, energy intake, REE) and Wilcoxon sum of ranks test (CRP). *P*-values reported have been corrected for the Bonferroni adjustment to allow for the multiple testing. Values for individual skinfold measurements are presented for completeness, but no statistical analyses were undertaken on these data.

In view of the range of weight changes within each of the three groups, *post hoc* correlation analyses were undertaken to examine the relationships between mean energy intake, mean REE and mean CRP (average of value prior to starting chemotherapy, prior to second chemotherapy and one month after the final chemotherapy treatment) and changes in weight and body composition (body fat, FFM and MAMC) within each of the three groups, using Pearson (energy intake, REE) and Spearman (CRP) correlation coefficients.

## RESULTS

### Patients

**Figure 1:**
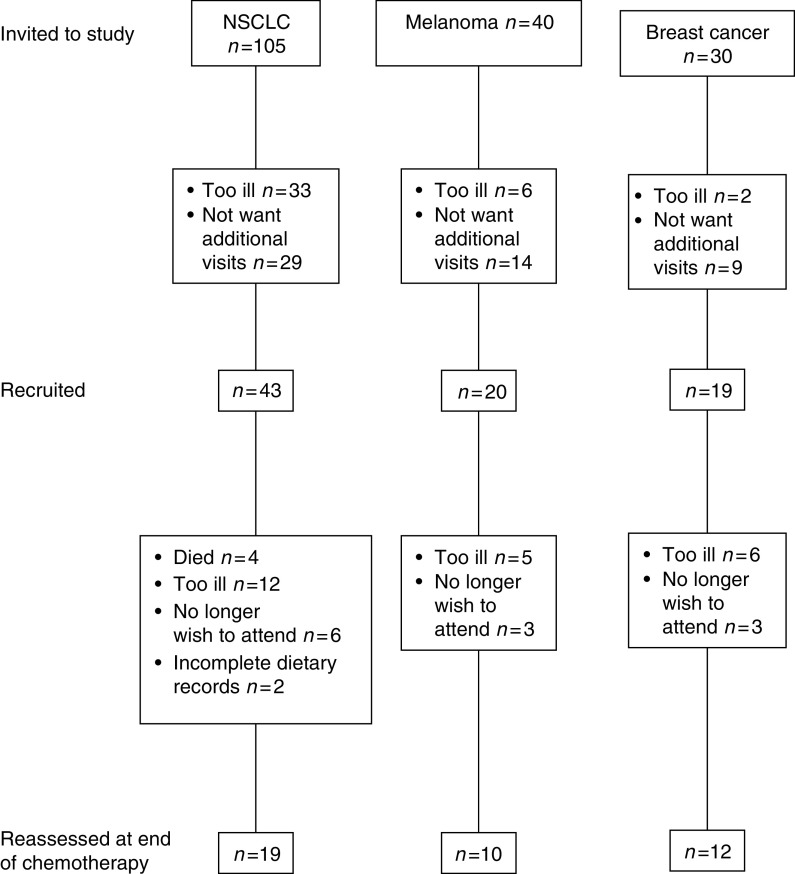
Recruitment and dropout of participants to study.

In all, 43 of the 105 patients with NSCLC, 20 of the 40 patients with metastatic melanoma and 19 of the 30 patients with metastatic breast cancer were recruited. There was a large dropout from the study. The reasons for not wishing to enter and for dropout from the study are shown in Figure 1. Results are presented for the 19 NSCLC, 12 melanoma and 10 breast cancer patients who were reassessed at the end of chemotherapy. All of the patients with NSCLC, four with breast cancer and 11 with melanoma were newly diagnosed with advanced disease. The remaining patients had metastatic disease, which had progressed after previous cancer therapy for advanced disease.

Baseline characteristics of patients recruited to the study and assessed throughout chemotherapy within the advanced NSCLC, melanoma and breast cancer groups are reported in Table 1 . Within the NSCLC and melanoma groups, patients assessed throughout chemotherapy were less likely to have an acute-phase protein response (APPR) at recruitment and more likely to have a favourable response to chemotherapy compared to the patients recruited the study. Non-small-cell lung cancer patients assessed throughout chemotherapy also had higher baseline performance scores than the group recruited to the study. In contrast, breast cancer patients assessed throughout chemotherapy were no more likely to have an APPR than the breast cancer patients recruited to the study, but if anything appeared less likely to have a favourable response to chemotherapy.

Among patients assessed throughout chemotherapy, those with NSCLC had experienced a significant weight loss in the 4 months prior to commencing chemotherapy. Median (range) weight change=−7(−18–7)%. Weight changes in the previous 4 months among patients with melanoma and breast cancer patients were not significantly different from zero; however, some of the patients within each group had lost or gained weight (Table 1). Chemotherapy cycles lasted for between 3 and 7 months: median (range) length of chemotherapy for patients with NSCLC=5 (3–7) months; metastatic melanoma=3.8 (3–6) months and metastatic breast=4.25 (4–6) months. A proportion of patients in each group responded to chemotherapy (objective response; seven NSCLC, one breast cancer, two melanoma, stable disease; three NSCLC, four breast cancer and four melanoma or progressive disease; nine NSCLC, five breast cancer and six melanoma). At 1 year after recruitment into the study, 13 of the 19 patients with NSCLC, eight of the 12 patients with melanoma and three of the 10 patients with breast cancer had died.

### Body mass and composition

Body mass and composition over the course of chemotherapy are reported in Table 2a . Mean weight did not change over the course of chemotherapy; however, some of the patients within each group had appreciable gains or losses of weight. Patients with metastatic breast cancer had a significant increase in % body fat mean change (95% CI)=2.1(0.8–3.5)% (*P*<0.05) and tendency to lose FFM; mean (95% CI change)=−1.9(−4.9–1.1) kg (*P*=0.074). Mean % body fat and FFM remained unchanged among patients with NSCLC and melanoma; however, some patients within each group had lost or gained body fat and FFM. Patients with metastatic melanoma had a significant decrease in MAMC mean (95% CI change)=−1(−2, −0.2) cm (*P*<0.05). Baseline and post chemotherapy TBK were assessed in 14/19 patients with NSCLC, 10/12 patients with metastatic melanoma and 8/10 patients with metastatic breast cancer. Mean TBK remained unchanged in each of the groups; however, patients within each of the groups had experienced increases or decreases in TBK.

### Energy intake and energy expenditure

Resting energy expenditure is expressed as absolute REE in terms of kJ/24 h, per kg, per kg FFM, and in order to standardise for weight, height and age as a % of the resting energy, expenditure was predicted from the Harris Benedict equation (% HB) (Harris and Benedict, 1919). Patients with NSCLC had a significantly higher REE compared to age- and sex-matched healthy subjects. REE for patients with NSCLC was 112 (17)% HB compared to 97.5% HB (10.4) in the controls (*P*<0.001). The REE in patients with metastatic melanoma was 104 (11)% HB, which was comparable to controls 101.4 (9)% HB (*P*=0.41). REE in patients with metastatic breast cancer was 98.6 (9.6)% HB, which was also comparable to controls 95.0(9.5)% HB (*P*=0.234).

Changes in REE and energy intake are reported in Table 2b. There were no significant overall changes in REE over the course of chemotherapy, but a variable response in REE within each of the three groups. Mean REE throughout chemotherapy did not relate to change in weight, FFM or body fat over the course of chemotherapy (data not shown).

**Figure 2:**
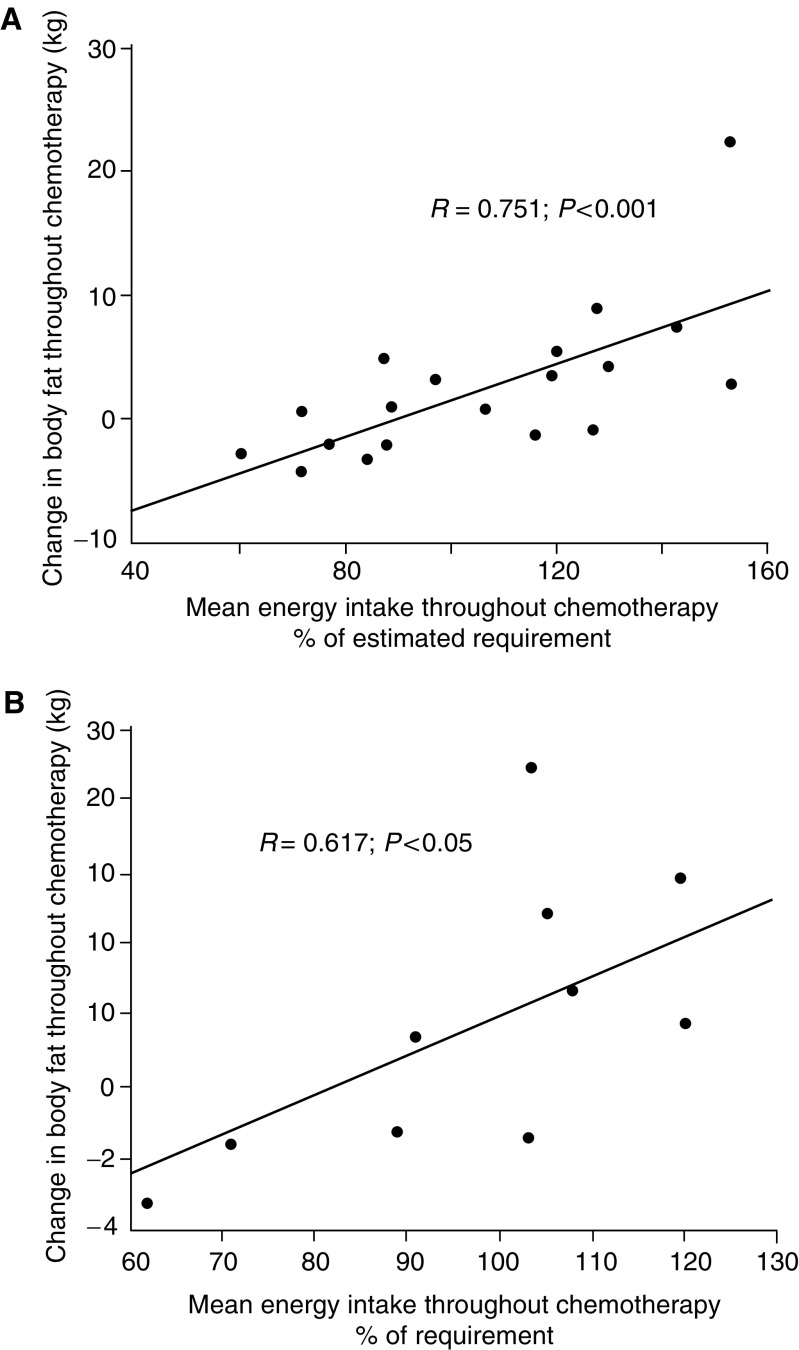
(**A**) Correlation between mean energy intake and change in body fat throughout chemotherapy among patients with NSCLC. (**B**) Correlation between mean energy intake and change in body fat throughout chemotherapy among patients with metastatic breast cancer.

Energy intake is expressed as an absolute intake in mJ and mJ per kg body weight. Patients with melanoma and breast cancer had significant increases in intakes of both energy and protein over the course of chemotherapy (*P*<0.05). Mean energy intake did not change for patients with NSCLC, but some patients had appreciable increases or decreases in energy intake over the course of chemotherapy. Correlations between mean energy intake throughout chemotherapy with change in weight, body fat and FFM in the three groups are shown in Table 3a . Mean energy intake throughout chemotherapy was significantly related to change in weight (*R*=0.603; *P*<0.01) and body fat (*R*=0.751; *P*<0.01) among patients with NSCLC (Figure 2A), and to the change in body fat among patients with breast cancer (*R*=0.617; *P*<0.05; Figure 2B). Gains in weight and body fat among patients with NSCLC and metastatic breast cancer are therefore related to higher energy intakes. Change in weight and body fat were not related to energy intake among patients with metastatic melanoma. Change in FFM and MAMC did not relate to energy intake in any of the groups.

### Health-related quality of life

Changes in FACT-G score are shown in Table 2a. Patients with metastatic breast cancer had a significant decrease in FACT-G score over the course of chemotherapy mean change (95% CI)=8 (−3,−12) (*P*<0.05). Mean FACT-G score remained unchanged among patients with NSCLC and metastatic melanoma.

### Inflammatory response

In all, 35 of the 43 patients recruited with NSCLC (82%), 15 of the 40 patients with melanoma (37%) and 13 of the 30 (44%) with breast cancer had an APPR defined as a CRP ⩾10 mg l^−1^ (Mahmoud and Rivera, 2002). Among patients assessed throughout chemotherapy, 11 of the 19 (56%) patients with NSCLC, two of the 12 with melanoma (18%) and four of the 10 (40%) with breast cancer had an APPR. There was no overall change in CRP over the course of chemotherapy in any of the groups. A higher proportion of the patients with melanoma had an APPR at the end of chemotherapy compared to baseline (4/12 compared to 2/12), while less metastatic breast cancer had an APPR at the end of chemotherapy compared to baseline (2/10 compared to 4/10). The proportion of patients with NSCLC with an APPR did not change between baseline and the end of chemotherapy (7/19 compared to 8/19).

Correlations between mean CRP over the course of chemotherapy and change in weight, body fat and FFM and MAMC are shown in Table 3b. Among patients with NSCLC, there were negative correlations between mean CRP and change in weight (*R*_s_ −0.549; *P*<0.05), body fat (*R*_s_ −0.543; *P*<0.05) and MAMC (*R*_s_ −0.577; *P*<0.05), but no relationship with change in FFM (*P*=0.799). Mean CRP level did not relate to changes in weight or body fat or FFM in patients with breast cancer or melanoma. Among patients with metastatic melanoma, there was a negative correlation between mean CRP and change in MAMC (*R*_s_ −0.617; *P*<0.05). The inflammatory response was therefore linked to loss of weight body fat and MAMC among patients with NSCLC and loss of MAMC among patients with melanoma.

## DISCUSSION

This study provides one of the few longitudinal assessments of energy balance (body composition, REE, dietary intake) and the systemic inflammatory response (APPR) among advanced cancer patients receiving chemotherapy. However, the high dropout rate from the study illustrates the problem of making serial measurements in such seriously ill populations. Selection bias is introduced among the patients with NSCLC and melanoma because patients in whom a second measurement could be made were probably among the less seriously ill, but not apparently among those with metastatic breast cancer.

We did not show the anticipated fall in body weight over the course of chemotherapy among patients with NSCLC or melanoma, but there was a range of nutritional responses to chemotherapy within these groups. Patients with breast cancer did not change the overall weight, but had a propensity to gain fat and to lose FFM. The ability to meet or exceed energy requirements led to gains in weight and body fat among patients with NSCLC and metastatic breast cancer, but did not prevent loss of FFM in either group. The acute-phase response was linked to loss of weight and body fat among patients with NSCLC and loss of MAMC among patients with NSCLC and metastatic melanoma.

Studies of wasting and cancer have mainly been conducted among patients with gastrointestinal (GI) malignancies (Bosaeus *et al*, 2001). We deliberately studied cancers that do not directly involve the GI tract to allow the effects of chemotherapy and the metabolic effect of the cancer being treated to be assessed. We have reconfirmed the variable change in weight among patients with NSCLC (Sarna *et al*, 1994). Weight loss has previously been reported among patients with metastatic melanoma (Smit *et al*, 1983). Weight maintenance in our patients may reflect less toxic chemotherapy regimens, and control of emesis. Loss of mid arm muscle alongside maintenance of FFM is these patients is consistent with previous reports of disproportionate loss of mid-arm muscle among cancer patients (Garrow, 1974). Advanced breast cancer patients are considered to maintain good nutritional status despite the presence of widespread metastatic disease (Bozzetti *et al*, 1981; Jardine *et al*, 1984), although not all studies concur (DeWys *et al*, 1981; Rowland-Payne *et al*, 1982). Gains in fat and loss of FFM among patients with breast cancer in this study represent a deterioration of nutritional status during chemotherapy.

We have confirmed the presence of an elevated REE among patients with NSCLC (Staal-van den Brekel *et al*, 1997a). Patients with both metastatic melanoma and metastatic breast cancer patients appeared to have a normal metabolic rate. There was a variable response in REE over the course of chemotherapy within each of the three groups. Previous reports have linked declines in REE with a response to chemotherapy (Jebb *et al*, 1994), the reasons for the varied response among patients in the current study are unclear.

Contrary to expectations, energy intake increased over the course of chemotherapy in patients with melanoma and breast cancer. This may in part reflect an effect of the pre-dose chemotherapy steroids among the breast cancer patients. Whether steroids would continue to influence dietary intake 3 weeks post treatment is not known. Increases in dietary intake were particularly surprising in the patients with metastatic melanoma since the majority of this population received highly emetogenic decarbazine chemotherapy and did not receive steroids. Chemotherapy-associated increases in dietary intake have previously been reported in patients with early but not metastatic breast cancer (Heasman *et al*, 1985). These increases are thought to be mediated by chemotherapy-associated hunger, an increased sense of well-being, or increased snacking to offset nausea (Grindel *et al*, 1989).

We have confirmed the prevalence of an APPR among patients with NSCLC at 82% (Scott *et al*, 2003). The APPR also occurred, but to a lesser extent, among patients with metastatic melanoma (37%) and metastatic breast cancer (44%). Patients taking anti-inflammatory medications, that is, steroids or ibuprofen, were included from the analysis as they still had an APPR. C-reactive protein would, if anything, be expected to be higher in these patients if they had not taken anti-inflammatory medications. Loss of weight and body fat was related to the level of CRP among patients with NSCLC, which confirms the findings from an earlier cross-sectional study (Scott *et al*, 1996).

The ability to meet or exceed energy requirements led to gains in body fat but not FFM in patients with breast cancer and NSCLC. This is consistent with findings from earlier nutritional intervention studies (Evans *et al*, 1987; Ovesen *et al*, 1993). Gains in fat have *not* been shown to confer advantages in terms of survival, chemotherapy-associated toxicity or quality of life among patients with NSCLC (Evans *et al*, 1987). The declines in health-related quality of life alongside gains in body fat among patients with metastatic breast cancer in the present study suggest that gains in fat were not beneficial in this group. Gains in body fat may be particularly deleterious for women with breast cancer. Higher weight and stores of body fat are linked to poorer survival and prognosis among patients with breast cancer (Petrelli *et al*, 2002; Borugian *et al*, 2003), most likely mediated by the associated higher levels of oestrogen (Leenen *et al*, 1994), insulin (Weinstock *et al*, 1998) and bioavailable insulin-like growth factor-1 (IGF-1) (Nam *et al*, 1997). Energy intake did not relate to changes in body fat among patients with metastatic melanoma in the present study. It is possible that the energy intake assessed 3 weeks after chemotherapy may not have reflected any acute decrease in energy intake in the immediate post-chemotherapy period. It also raises the possibility that changes in fat mass among patients with metastatic melanoma may be independent of energy balance and instead influenced by the presence of lipid-mobilising factors such as zinc-*α* 2 glycoprotein (Russell and Tisdale, 2002; Bing *et al*, 2004).

Preservation of FFM remains a major therapeutic challenge in the management of the cancer patients linked to improvements in functional capacity, prognosis (Tisdale, 2001), and quality of life (Fearon *et al*, 2003). Our study highlights that adequate dietary intake will not lead to gains in FFM during chemotherapy. Previous studies have linked loss of FFM (Simons *et al*, 1999; Fearon *et al*, 2003) to the presence of an inflammatory response in advanced cancer patients not receiving active treatment. We were unable to establish any links between CRP level and change in FFM in any of our advanced cancer groups over the course of chemotherapy. Loss of mid-arm muscle was linked to CRP among patients with NSCLC and metastatic melanoma, suggesting that loss of peripheral muscle mass at this site was linked to the inflammatory response. Total body potassium is considered by some to be a robust method of assessing lean body mass among cancer patients (Mcmillan *et al*, 2000). We, however, failed to replicate a previous report that declines in TBK in cancer patients are linked to the APPR (Mcmillan *et al*, 1998) (data not shown). A major distinction between the present and earlier reports that linked loss of FFM and TBK to the APPR is that our cancer patients were assessed throughout chemotherapy. Loss of FFM and TBK in patients receiving chemotherapy may be a function of the chemotherapy itself, which may limit protein synthesis (Herrmann *et al*, 1981; Tayek and Chlebowski, 1992), or may relate to the associated lower levels of hormones such as testosterone (Aaesebo *et al*, 1991) and IGF-1 (Kajdaniuk and Marek, 2000). The use of predose steroids among patients with NSCLC and breast cancer is unlikely to have led to loss of FFM in the doses used (Picado *et al*, 1990). Declines in activity (secondary to chemotherapy associated fatigue) may be a specifically linked to loss of FFM among advanced cancer patients receiving chemotherapy. We did not assess physical activity, but preliminary studies among advanced cancer patients receiving chemotherapy have demonstrated home-based resistance and moderate exercise programmes to be efficacious in the preservation of FFM (Coleman *et al*, 2003).

The possible limitation of assessment of FFM from skinfolds needs to be considered. No method of assessing lean (metabolically active tissue) is entirely satisfactory, particularly among cancer patients. All techniques (body potassium, dual emission X-ray absorptiometry, body water, body density, body impedance, *in vitro* neutron activation analysis (IVNAA)) make assumptions about the relationship between the various body compartments and what is being measured (Burkinshaw, 1990). FFM calculated from skinfold relies on a relationship with body density (Durnin and Womersley, 1974). It is, however, the most widely used and the most robust of the techniques available. Hill *et al* (1978) demonstrated a very close relationship between FFM and total body nitrogen (protein) measured using IVNAA. Mid upper arm muscle circumference is often used as an index of lean or fat-free body mass, but in wasting conditions tends to diminish faster than total body FFM (Garrow, 1974). Total body potassium is considered to be an index of the functional body cell mass and muscle mass (Moore, 1980). Previous studies have shown declines of TBK in patients receiving chemotherapy with a variety of tumours (Cohn *et al*, 1982) and testicular cancer (Hyltander *et al*, 1991). The reason for the lack of decline in TBK over the course of chemotherapy in patients in this study is unclear.

Small numbers of patients means that we were unable to assess whether response to chemotherapy influenced food intake, REE, body composition or APPR in each of the cancer groups. Response to chemotherapy has been linked to declines in REE, and preservation of FFM among patients with SCLC (Jebb *et al*, 1994) and declines in APPR (Staal-van den Brekel *et al*, 1997b). Some of the differences in body composition between the cancer groups may be in part due to gender effects (Harvie *et al*, 2003), but we are unable to determine these gender effects from our analysis.

This study adds to the body of data that shows the varied nutritional response to chemotherapy even within individuals with the same tumour stage. Higher energy intakes lead to gains in fat among patients with advanced breast and lung cancer. Loss of FFM throughout chemotherapy, however, does not appear to be linked to inadequate intakes of energy or protein. Declines in MAMC were linked to the presence of an APPR. Preservation of FFM alongside chemotherapy treatments may not therefore be amenable to standard nutritional repletion. Further studies should investigate the potential benefits of anti-inflammatory medications (Fearon *et al*, 2003) and exercise (Coleman *et al*, 2003) on preservation of FFM among advanced cancer patients receiving chemotherapy.

## Figures and Tables

**Table 1:** Baseline characteristics of patients with advanced NSCLC, metastatic melanoma and metastatic breast cancer recruited to study and assessed t hroughout chemotherapy

	**Advanced NSCLC**		**Metastatic melanoma**		**Metastatic breast cancer**	
	**Recruited 28 men, 15 women**	**Assessed 15 men, four women**	**Recruited 13 men, seven women**	**Assessed nine men, three women**	**Recruited 20 women**	**Assessed 10 women**
Age (years)	59.5 (8.5)^a^	59.0 (7.1)	55.9 (9.2)	54.4 (7.1)	52.4 (9.7)	55.9 (6.5)^a^
Weight change over the previous 4 months (%)	−7 (−27–16)^b^	−7 (−18–7)	−2.9 (−1–6.0)	−2.5 (−13–11)	8.5 (−27–27)	3 (−15–12)
BMI (kg m^−2^)	24.1 (4.3)	24.7 (4.5)	25.6 (3.0)	26.5 (2.7)	28.4 (8.5)	29.1 (7.2)
Smoker	38%	36%	25%	50%	20%	0%
Presence of APPR (%)^c^	82%	56%	37%	18%	44%	40%
Karnofsky score	78 (11)	84 (10)	86 (11)	88 (12)	87.3 (12.4)	88 (12.5)

*Medications (n)*						
Ibuprofen	5	2	2	2	2	0
Megace	3	2	0	0	0	2

*Steroids*						
Predose with chemotherapy^d^	36	17	0	0	19	10
Daily prednisolone	15	4	2	0	1	0

*Response to chemotherapy*						
Objective response	22%	37%	20%	17%	38%	10%
Stable disease	13%	16%	20%	33%	31%	40%
Progressive disease	65%	47%	60%	50%	31%	50%

a Mean (s.d.).

b Median (range).

c The presence of an acute-phase protein response defined as CRP>10 mg l^−1^ (Mahmoud *et al*, 2002).

d Predose of dexamethasone 8–40 mg for 3–5 days with chemotherapy.

**Table 2:** (a) Change in body mass and composition and health-related quality of life and (b) resting energy expenditure dietary intake and C-reactive protein over the course of chemotherapy in patients with advanced NSCLC, metastatic breast cancer and metastatic melanoma

	**Advanced NSCLC: 15 males, four females**		**Metastatic melanoma: nine males, three females**		**Metastatic breast cancer: 10 females**	
	**Prechemotherapy**	**Δ from Prechemotherapy**	**Prechemotherapy**	**Δ from Prechemotherapy**	**Prechemotherapy**	**Δ from Prechemotherapy**
(*a*)						
Weight (kg)	72.8 (17.9)^a^	0.94 (−3,4.9)^b^	81.0 (13.7)	0.24 (−2.4, 2.9)	73.6 (18.9)	−0.3 (−5.7,5.1)
FFM (kg)	52.6 (11.6)	−1.1 (−3.1,0.9)	56.2 (9.8)	0.6 (−1.6, 2.9)	45.7 (8.9)	−1.9 (−4.9,1.1)
Total fat (kg)	20.1 (8.5)	2.1 (−1.2,5.3)	24.7 (6.6)	−0.6 (−3.4, 2.2)	27.9 (10.7)	1.5 (−1.2,4.4)
% Body fat	26.9 (6.8)	1.7 (−0.9, 4.3)	30.4 (5.5)	0.36 (−3.6, 2.9)	37.3 (5.0)	2.1 (0.8, 3.5)*
Mid arm muscle circumference (cm)	25 (3.2)	0.0 (−0.9,0.9)	28.5 (2.4)	−1 (−2,−0.2)*	28 (2.0)	−1 (−2.3−0.4)


Total body potassium (counts)^c^	1115 (116)	8.0 (−67–82)	1252 (210)	−99 (−235,35)	856 (189)	65 (−54,186)
Tricep skin fold (mm)	11.1 (4.2)	1.0 (−1.7, 3.6)	12.7 (4.6)	−0.2 (−3.4, 3)	16.0 (11.0)	1.8 (−0.3−4.0)
Bicep skinfold (mm)	7.4 (3.5)	0.4 (−2.5,3.3)	8.9 (3.4)	−0.7 (−3.5,2.1)	10.0 (7.0)	0.7 (−3,5)
Subscapular skin fold (mm)	18.0 (8.3)	3.3 (−1.6–8.2)	21.6 (8.0)	1.4 (−3.5,6.3)	29.0 (12.3)	3.3 (−3.3,8.6)
Suprailiac skinfold (mm)	15 (10)	3.7 (−0.5–8.0)	210.5 (9.0)	−2 (−9,5)	30.0 (16.1)	5.6 (−0.7,12.0)
Fact-G score	86 (15)	−3 (−14,9)	89 (20)	0.8 (−7, 8)	91 (14)	−8 (−3,12)*

(*b*)						
REE (kJ)	7250 (1807)	−334 (−874, 205)	7217 (1380)	66 (−418,548)	5887 (1334)	15 (−297,170)
REE (kJ kg^−1^)	100 (19)	−5.9 (−8.8.34)	89 (4.6)	0 (−7,8)	78 (6.7)	0.4 (−10.5,10.5)
REE (kJ kg^−1^ FFM)	138 (21)	−3 (−11,5.5)	129 (11)	−0.8 (−12,10)	129 (12)	5 (−0.1,11)
Energy intake (kJ)	10439 (3565)	381 (−1209,1970)	7246 (2004)	600 (125,1076)	7552 (1840)	974(326,1230)*
Energy intake (kJ kg^−1^)	154 (66)	1.3 (−23,26)	96 (25)	30 (8,50)	100 (3.1)	10 (4,17)*
Protein intake (g)	98.4 (33)	−3.5 (−17, 10)	72.3 (15)	22.5 (5.0,40.5)	71.3 (23.4)	7.3 (1.3,13)*
C-reactive protein (mg l^−1^)	17.5 (5–182)^d^	−0.3 (−31,31)	5 (5, 24)	13.6 (−4.3, 31.6)	6.5 (5,165)	2.4 (−20, 25)

a Mean (s.d.)

b Mean (95% confidence intervals).

c NSCLC *n*=14; metastatic melanoma *n*=10; metastatic breast cancer *n*=8.

d Median (range). FFM=fat-free mass; REE=resting energy expenditure.

* Significant difference between prechemotherapy and post chemotherapy (*P*<0.05).

* Significant difference between prechemotherapy and post chemotherapy (*P*<0.01).

**Table 3:** (a) Pearson correlations between mean energy intake and (b) Spearman correlation between mean level of C-reactive protein over the course of chemotherapy and change in weight, fat, fat-free mass and mid-arm muscle circumference over the course of chemotherapy in advanced cancer patients

	**Change in weight**	**Change in total body fat**	**Change in fat-free mass**	**Change in mid-arm muscle circumference**
*(a)*				
NSCLC *n*=19	0.603*	0.751*	−0.213	−0.196
Metastatic melanoma *n*=12	0.427	−0.112	0.329	0.259
Metastatic breast cancer *n*=10	0.533	0.617*	0.383	−0.050

*(b)*				
NSCLC *n*=19	−0.549*	−0.543*	−0.069	−0.577*
Metastatic melanoma *n*=12	0.098	−0.328	−0.160	−0.617^*^
Metastatic breast cancer *n*=10	−0.018	0.162	0.108	0.117

* Correlation is significant at the 0.05 level (two-tailed).

* Correlation is significant at the 0.01 level (two-tailed).
